# Supplementary material for: A new kind of 2D topological insulators BiCN with a giant gap and its substrate effects
Source: Sci Rep. 2016 Jul 21;6:30003. doi: 10.1038/srep30003 (PMC4956758; doi:10.1038/srep30003)
Supplement: Supplementary Information [file srep30003-s1.pdf]

Supplemental Material for "A new kind of 2D topological insulator BiCN with a giant gap and its substrate effects"

Botao Fu,[1] Yanfeng Ge,[1] Wenyong Su,[1] Wei Guo,[1] and Cheng-Cheng Liu[1,*]

[1]*School of Physics, Beijing Institute of Technology, Beijing 100081, China*

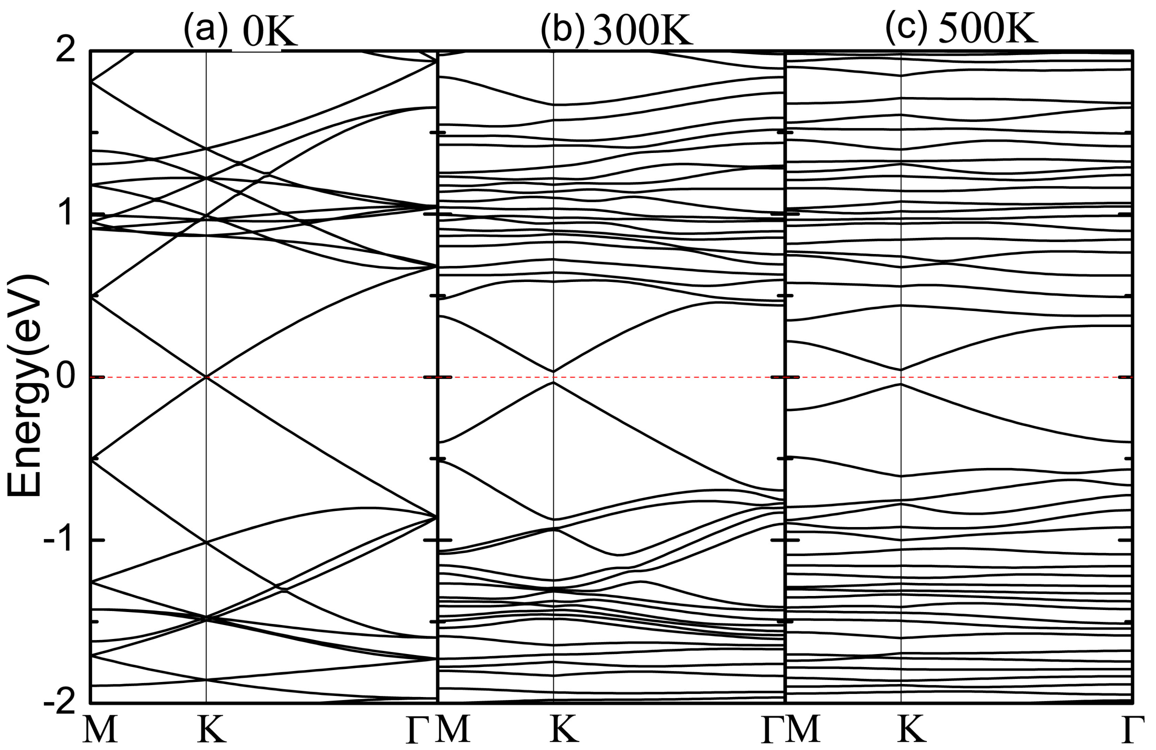

FIG. 1. Bandstructure without SOC of BiCN 4×4 supercell before MD stimulation (a), after MD stimulation for 2.3 ps at 300K (b) and 500K (c).

I. THE ELECTRONIC STRUCTURE OF BICN AT FINITE TEMPERATURE

The MD stimulation is performed for the 4×4 supercell of BiCN monolayer with time step of 1.5 fs. In our main text we have shown the structure is stable even at 500K. Here in Fig. 1, we calculate the bandstructures of BiCN at 300K and 500K. Comparing the bandstructures at 0K, 300K, and 500K in Fig.1, we notice there are obviously band degeneracy lifting above 0.5 eV away from Fermi Level due to the reduced symmetry of the finite temperature structures. However we find the bands near Fermi level have very little change except a small gap openning at K points, due to the structural thermal perturbation at finite temperature. The size of the band gap at K point is about 62 meV and 81 meV for 300K and 500K respectively, which is one order smaller than the SOC induced gap(1.28 eV). Thus, for BiCN, both the structure and the topological property are robust at 300K and 500K, indicating BiCN can be a much promising candidate for realization of room temperature quantum spin Hall insulators.

II. THE ELECTRONIC STRUCTURE OF BICN UNDER STRAIN AND EXTERNAL ELECTRIC FIELD

Strain and external electric effects are usually inevitable for 2D materials on certain substrates, which possibly affect their intrinsic properties[1–3]. Therefore we simulate the biaxial in-plane strain effects simply by changing the optimized lattice constant and external electric fields by imposing a perpendicular electric field. Fig. 2(a) shows the bandstructures of BiCN when the in-plane biaxial strain varies from -6% to 6%. First we can see the band edges and band gap around K point change very little because the band gap is determined by the atomic SOC. On the other hand, the bands around M point and valence bands around Γ point show remarkable dependence on strain. Since the VBM at K and CBM at Γ are rather robust against the strain, the indirect gap keeps almost the same. Fig. 2(b) displays the evolution of bandstructure around K for BiCN when the external electric fields varies from 0 to 0.6 V/. The main features are the spin splitting of both CBM and VBM due to the breaking down of the inversion symmetry at the present of an electric field. The band gap gradually decreases with the increase of electric field with the ratio of 0.12 e. Due to the extremely large SOC bulk gap and the nearly coplanar Bi atoms in BiCN, 0.4 V/ can only decrease the gap by about 50 meV, 4.2% of its intrinsic gap at K. Therefore, we can conclude that the BiCN is rather

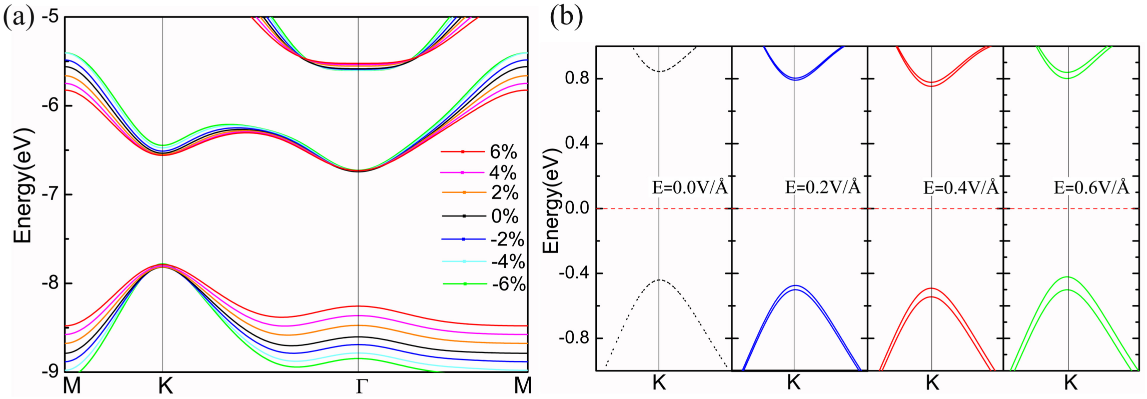

FIG. 2. Bandstructure under strain and electric fields. (a) Bandstructures of BiCN under different biaxial in-plane strain. Here $\varepsilon = (a - a_0)/a_0$, where a_0 is the optimized lattice constant. The positive and negative values of ε indicate the biaxial tensile and compressive strain respectively. The energy is aligned with respect to the vacuum level. (b) Bandstructures of BiCN around K point under different vertical external electric fields.

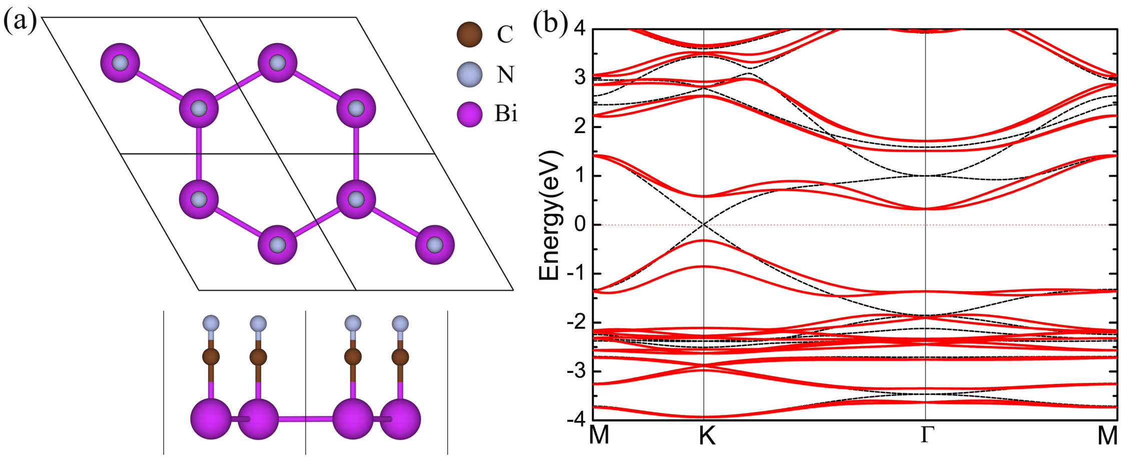

FIG. 3. Geometric and electronic structure for $\beta-$BiCN. (a) Geometric structure of $\beta-$BiCN from top and side views. (b) The bandstructure of intrinsic $\beta-$BiCN with SOC (red line) and without SOC (black line).

robust 2D TI against the strain and external electrical field.

III. THE GEOMETRIC AND ELECTRONIC STRUCTURE OF $\beta-$BICN

Here another 2D material β-BiCN (an allotrope of the symmetric BiCN) is shown in Fig. 3(a). $\beta-$BiCN can be regarded as a bilayer Bi film passivated by -CN from oneside. We propose that this structure is possibly realized by growing bi-layer Bi on substrates in experiment, and then processing with chemical modification. The optimized lattice constant of $\beta-$BiCN is 5.77 , which is 4.2% larger than BiCN. All the Bi atoms lie in the same plane with the nearest neighboring distance of 3.33 . The bandstructure of $\beta-$BiCN with and without SOC are shown in Fig. 3(b). Similar to BiCN, $\beta-$BiCN also has a large direct band gap about 0.90 eV opened by SOC at K. The indirect gap is 0.64 eV, from VBM at K and CBM at Γ. The topological properties of $\beta-$BiCN are also consistent with BiCN, a 2D TI. Besides, we find there is a remarkable spin splitting at the valence band around K point due to the absence of space inversion symmetry. The splitting energy is as large as 0.53 eV. This may be very helpful for spintronics applications.

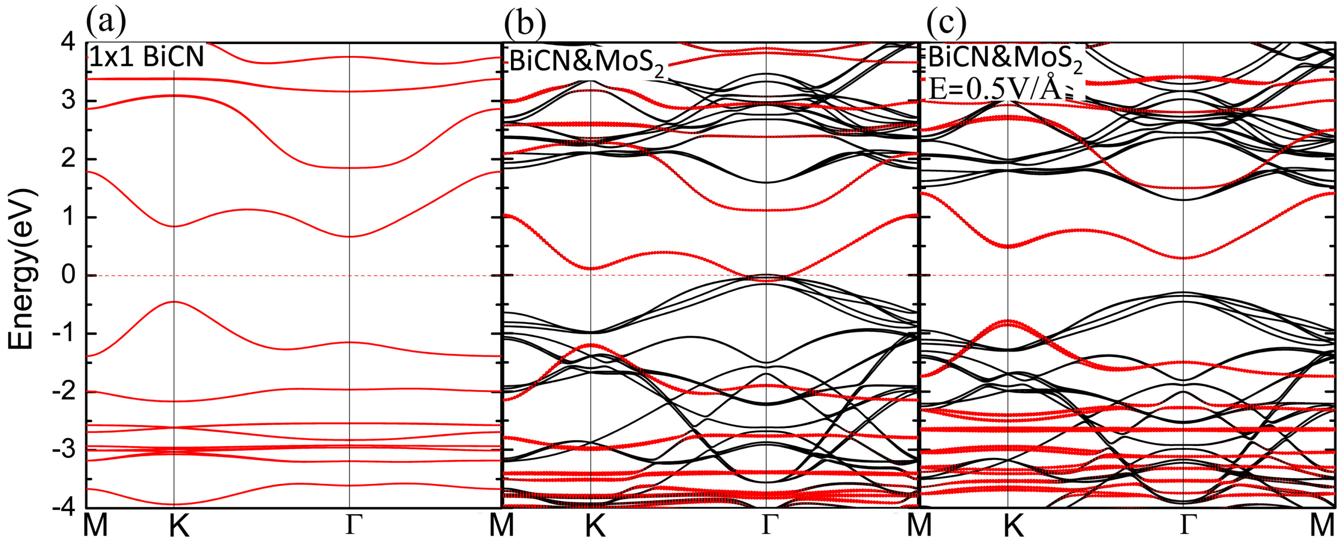

FIG. 4. Bandstructure for BiCN on MoS_2. (a) Bandstructures for 1×1 BiCN, (b) Bandstructure of 1×1 BiCN on $\sqrt{3} \times \sqrt{3}$ MoS_2, (c) Bandstructure of 1×1 BiCN on $\sqrt{3} \times \sqrt{3}$ MoS_2 with a vertical electric field E=0.5 V/. The electric field points from substrate MoS_2 to BiCN.

IV. THE MANIPULATION OF BANDSTRUCTURES OF VDW HETEROJUCTIONS BY AN EXTERNAL ELECTRIC FIELD

The electronic properties of vdW heterojuctions are greatly determined by the work function difference of each component except for their individual properties[4]. As schematically shown in Fig. 4, due to a large difference of the work function of BiCN (7.4 eV) and substrates (5.2 eV), the CBM of pristine BiCN lies below the VBM of pristine MoS_2, which makes the composite system to be metallic, in spite of the fact that its constituents are both insulating. This is similar with the picture of ON state of typical tunnel field-effect transistor (TFET)[5,6],where the carriers in VBM of source material may tunnel into the CMB channel material, depending on the middle barrier height and width between them. The metallic nature of this vdW heterojuction is unwished for TIs. We here address this issue by applying a vertical gate voltage, which takes advantage of the structure feature of the composite system. In the right part of Fig. 4, we can see, under a positive electric field, the band of BiCN can be shifted up respective to MoS_2 to recover the insulating state as a whole. This proposal is further supported by our first principle calculations in Fig. 3. In the Fig. 3(b), the Fermi level of the composite system just crosses the VBM of h-BN layer and CMB of MoS2, means it's metallic. In Fig. S3(c) while imposing a vertical electric field of E=0.5 V/ directed from MoS_2 to BiCN, the band can be tuned effectively, as expected. Therefore this composite system can well maintain topological and insulating at the same time.

* ccliu@bit.edu.cn
1 Huang, Z.-Q. *et al.*. Nontrivial topological electronic structures in a single Bi(111) bilayer on different substrates: A first-principles study. *Phys. Rev. B* **88**, 165301(2013).
2 Ni, Z. H. *et al.* Raman spectroscopy of epitaxial graphene on a SiC substrate. *Phys. Rev. B* **77** 115416 (2008)
3 Zhu, F.-F. *et al.* Epitaxial growth of two-dimensional stanene. *Nat. Mat.* **14**, 1020 (2015)
4 Ozcelik, V. O., Azadani, J. G., Yang, C., Koester, S. J. & Low, T. Band Alignment of 2D Semiconductors for Designing Heterostructures with Momentum Space Matching. arXiv: 1603.02619 (2016)
5 Sarkar, D. *et al.* A subthermionic tunnel field-effect transistor with an atomically thin channel. *Nature* **526**, 91-95 (2015)
6 Ionescu, A. M. & Riel, H. Tunnel field-effect transistors as energy-efficient electronic switches. *Nature* **479**, 329-337 (2011)

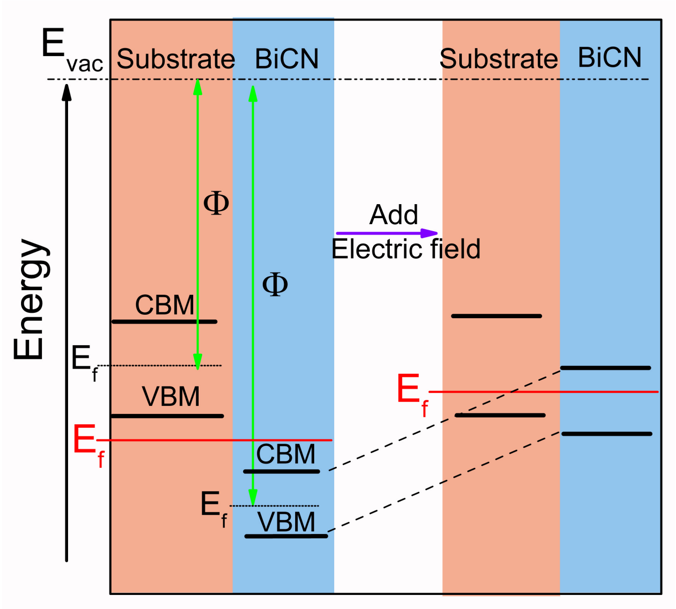

FIG. 5. Schematic diagram for band manipulation by an external electric field. From left side to right side, it shows the positions of band edges of BiCN and substrates before and after adding electric field. The black dash line at the top means the vacuum level E_{vac}, and the black dot lines between CBM and VBM indicate the Fermi level of substrate and BiCN separately. The red solid lines indicate the Fermi level positions for the composite systems. The symbol Φ represents the work function of BiCN and substrate.
